# 

**Published:** 1979

**Authors:** 


JANUARY/APRIL 1979

VOLUME 94 (i/ii)

NUMBER 349/350

Published Quarterly

Annual Subscription £6 Post Free

### BRISTOL MEDICO-CHIRURGICAL JOURNAL

Contents

3 The Evolution of Medical Societies in Britain — Have They a Future?

Donald S. Ractliffe

Obituary

11 W. G. J. Hampson, M.A., B.M., B.Ch., F.R.C.S.Ed.

12 Dr. Clifford D. Evans

Established 1883

### BRISTOL MEDICO-CHIRURGICAL JOURNAL

Incorporating the Medical Journal of the South West

### Editorial Committee

EDITOR
*Dr. D. S. Reeves*
*Southmead Hospital, Bristol, BS10 5NB*
ASSISTANT EDITOR
*Dr. A. P. Radford*
MEMBERS
*Dr. Rhys Davies*
*Dr. M. B. Lennard*
*Dr. D. W. Wright*
*The Hon. Treasurer of the Society*
*The Hon. Secretary of the Society*
SECRETARY
*Mr. B. P. Jones, The Library, Medical School,*
*University Walk, Bristol, BS8 1TD*

### Bristol Medico-Chirurgical Society

PRESIDENT 1978/79
*H. K. Lucas, Esq.*
PRESIDENT ELECT
*Dr. D. S. Ractliffe*
HONORARY SECRETARY
*A. J. Webb, Esq., 7 Percival Road, Bristol, BS8 3LE*
HONORARY TREASURER
*Dr. J. Penry, Southmead Hospital, Bristol, BS10 5NB*

The Society was founded in 1874. In 1883 publication of the Journal began and has continued quarterly ever since then. During the years 1949 to 1962 it appeared under the title of 'The Medical Journal of the South West'.

### Notice to Contributors

*The Journal is especially concerned to provide a place for the recording of medical thought, interests, and practice in and around Bristol. It therefore welcomes articles that, as well as being of immediate interest to members, will document the local and contemporary medical scene for our successors.*

*Original articles are invited provided that they have not been published elsewhere. Illustrations should be supplied ready for reproduction. Line drawings should be in Indian ink on firm white paper or board. The author will be informed if it is found necessary to ask him to pay for the printing of photographs etc.*

*The following contributions will be especially welcome; notes of forthcoming events, meetings held, degrees conferred, staff appointments, honours and public appointments and other local news.*

*Advice on preparation can be obtained from the Editor.*

Printed by the University of Bristol Printing Unit

### University of Bristol

### NEW YEAR HONOURS, 1979

Dr. Sheila Fraser, O.B.E.

### FACULTY OF MEDICINE

### The Degree of Doctor of Philosophy
**January 1979**

Ian Colin BARNES
Krystyna Franciszka JABLONSKA
Stephen John William LISNEY
Stephen NICKLIN
Philip SLOAN

**March 1979**

Ahmed Mohamed HUSSEIN

### The Degree of Doctor of Medicine
**January 1979**

Mamoun Mohamed Ali HOMEIDA
Kundurty Purnananda SARMA

**March 1979**

Suliman Salih FEDAIL
Hassan Musa ISMAIL

### The Degree of Master of Science in Medical Sciences
**January 1979**

Mohammed Ashraf HUSSAIN
Abdul-Aziz KHEZRI
Robin PHILIPP

**March 1979**

Ian Alexander STEWART SCOTT

### The Degree of Master of Dental Surgery
**January 1979**

Roger Grenville SMITH

### Final Examination for the Degree of B.D.S. (Section II)
**November/December 1978**

**WITH HONOURS**

Judith Margaret Ismae ATKINSON
Maria Margarita Equagoo COCKLE
and with Distinction in Section II
Christopher Alan GOVER
and with Distinction in Section II
Margaret Anne SOFTLY
and with Distinction in Section II
Stephen WATTS

**PASS**

Kevin Charles ABBISS
Ingrid Christine ARNTZEN
Sharon Denise BENNETT
Christine Louise BENTLEY
Pravinchandra Ramjibhai BHOGAITA
Simon King BLACKBURN
Colin Richard BUNCE
Bernadette Christine CARNE
Catherine Sarah CROMPTON
Jennifer Sian DAVIES
Robert Michael Anthony FIRTH
Nichola Annemarie HAWKINS
Jacqueline Ann HEMMINGS
Jonathan Frederick HICKS
Pamela Ann JACKSON
Carl JOHNSON
Jacqueline Margaret JONES
Duncan Garvie LAMOND
Donald Andrew LANE
Marc Samuel LEVY-BENCHETTON
Tori Helene LUNDEBY
Peter MAGUIRE
Sailesh R. PATEL
Jane Caroline POLGE
Sushil Kumar M. RAJPAL
Mary Frances ROBSON
Janet Fiona SCOTT
Mark Andrew SMITH
Sudhir Natwarlal THAKERAR
Ikbal Kassamali VIRJI
Sarah Jane WILLIAMS
John Michael WORSLEY
Caroline Jane WORTELHOCK
Paul Graham WYLIE

### A SELECTION OF RECENT ADDITIONS TO THE MEDICAL LIBRARY

*Continued from:* **July/October 1978 Vol.93(iii/iv) No.347/348**

Neale, A. V. et al. The care of the premature baby in the Bristol area: a symposium. Southmead Gen.Hosp.Gp.Management Comm. (c.1952).

NIEBURGS, H. E. The prevention and detection of cancer. Pt. 1: Prevention. Vol. 1: Etiology. Dekker.

NISHIMURA, H. and OKAMOTO, N. Sequential atlas of human congenital malformations. Igaku Shoin 1976.

OAKLEY, W. G. et al. Diabetes and its management. Ed. 3. Blackwell.

O'MALLEY, B. W. and BIRNBAUMER, L. Receptors and hormone action. Vol. 1. Academic.

OXFORD, J. S. Chemoprophylaxis and virus infections of the respiratory tract. Vol. 1. CRC Press.

Oxford-Harrap Standard German-English Dictionary. Vols. 1—3, A—R, Clarendon Pr.

PARR, J. Introduction to ophthalmology. Oxford UP.

PHILLIPS, I. et al. Microbiological hazards of infusion therapy. MTP. 1976.

PINCUE, L. and DARE, G. Secrets in the family. Faber.

PUFFER, R. R. Familial susceptibility to tuberculosis: its importance as a public health problem. Harvard UP. 1944.

RAINS, A. J. H. 1001 multiple choice questions and answers in surgery, H. K. Lewis.

RAJKA, E. and KOROSSY, S. Immunological aspects of allergy and allergic diseases. Vol. 3: clinical aspects of autoimmune diseases. Plenum. 1975.

REIVICH, M. et al. Tissue hypoxia and ischemia. Plenum.

Reviews of Physiology, Biochemistry and Pharmacology. Vol. 84. Springer.

ROBINSON, D. Patients, practitioners and medical care: aspects of medical sociology. Ed. 2. Heinemann.

Royal College of Psychiatrists. Education Committee. Handbook for inceptors and trainees in psychiatry. (ed.) T. Bewley. Headley.

RUSSELL, P. M. G. A history of the Exeter hospitals 1170—1948. Exeter PMC.

SALMON, M. A. and LINDENBAUM, R. H. Developmental defects and syndromes. HM & M.

SAMMAN, P. D. Nails in disease. Ed. 3. Heinemann.

SANDLER, M. Mental illness in pregnancy and in the puerperium. Oxford UP.

SATIR, V. Conjoint family therapy: a guide to theory and technique. Souvenir Pr.

SAWYER, P. N. and KAPLITT, M. J. Vascular grafts. ACC.

SCHEBITZ, H. and WILKENS, H. Atlas of radiographic anatomy of the horse. Ed. 3. Vlg.Paul Parey.

SCHÖNDORF, H. Aspiration cytology of the breast. Transl. by V. Schneider. Saunders.

SCHOENFIELD, L. J. Diseases of the gallbladder and biliary system. Wiley.

SCOTT, A. C. Neurophysics. Wiley.

SCOTT, J. H. and DIXON, A. D. Anatomy for students of dentistry. Ed. 4. Churchill Livingstone.

SHIRLEY, I. M. et al. A user's guide to diagnostic ultrasound. Pitman.

SILVERSTONE, T. and TURNER, P. Drug treatment in psychiatry. Ed.2. Routledge.

SKOLNICK, A. The intimate environment: exploring marriage and the family. Little, Brown. 1973.

SKRABANEK, P. and POWELL, D. Substance P. Vol. 1. Churchill Livingstone.

SNEDECOR, G. W. and COCHRAN, W. G. Statistical methods. Ed. 6. Iowa State Univ.Pr.

SNELL, R. S. Cross anatomy dissector: a companion for Atlas of clinical anatomy. Little, Brown.

Society of Medical Officers of Health. The functions of the medical officer of health. SMOH (1955).

STELL, P. M. and MARAN, A, G, D. Head and neck surgery. Ed. 2. Heinemann.

Subcellular Biochemistry. Vol. 5 (1978). Plenum.

TODD, I. P. Intestinal stomas. Heinemann.

TUNNADINE, D. and GREEN, R. H. Unwanted pregnancy — accident or illness? Clarendon Pr.

TURNER-WARWICK, M. Immunology of the lung. Arnold.

ULLRICH, V. et al. Microsomes and drug oxidations. (Suppl. to 'Biochemical pharmacology') Pergamon.

VICKERS, M. D. et al. Drugs in anaesthetic practice. Ed. 5. Butterworth.

VIDA, J. A. Anticonvulsants (Medicinal Chemistry, 15) Academic.

VOELLER, B. R. The chromosome theory of inheritance. Plenum.

WATKIN, B. The National Health Service: the first phase 1948—1974 and after. Allen & Unwin.

WATKINS, W. B. Hypothalamic releasing factors. Vol. 2. Churchill Livingstone.

WEIR, J. and ABRAHAMS, P. An atlas of radiological anatomy. Pitman.

WEXLER, D. A. and RICE, L. N. Innovations in client-centred therapy. Wiley.

WILLOCKS, J. Essential obstetrics and gynaecology: a guide for postgraduates. Churchill Livingstone.

Women's Group on Public Welfare. Hygiene Committee. Our towns: a close-up. Oxford UP. 1943.

WYLIE, W. D. and CHURCHILL-DAVIDSON, H. C. A practice of anaesthesia. Ed. 4, by H. C. Churchill-Davidson. Lloyd-Luke.

YABLONSKY, L. The tunnel back: Synanon. Macmillan 1965 repr. 1967.

Year Book of Cardiology, 1978. Year Bk.Med.Publ.

Year Book of Otolaryngology, 1978. Year Bk.Med.Publ.

